# Update on the Applications of Radiomics in Diagnosis, Staging, and Recurrence of Intrahepatic Cholangiocarcinoma

**DOI:** 10.3390/diagnostics13081488

**Published:** 2023-04-20

**Authors:** Maria Chiara Brunese, Maria Rita Fantozzi, Roberta Fusco, Federica De Muzio, Michela Gabelloni, Ginevra Danti, Alessandra Borgheresi, Pierpaolo Palumbo, Federico Bruno, Nicoletta Gandolfo, Andrea Giovagnoni, Vittorio Miele, Antonio Barile, Vincenza Granata

**Affiliations:** 1 Department of Medicine and Health Sciences “V. Tiberio”, University of Molise, 86100 Campobasso, Italy; 2 Clinical Pharmacology Unit, A. Cardarelli Hospital, 86100 Campobasso, Italy; 3 Medical Oncology Division, Igea SpA, 80013 Naples, Italy; 4 Nuclear Medicine Unit, Department of Translational Research, University of Pisa, 56126 Pisa, Italy; 5 Italian Society of Medical and Interventional Radiology (SIRM), SIRM Foundation, Via della Signora 2, 20122 Milan, Italy; 6 Department of Emergency Radiology, Careggi University Hospital, Largo Brambilla 3, 50134 Florence, Italy; 7 Department of Radiology, University Hospital “Azienda Ospedaliera Universitaria delle Marche”, 60121 Ancona, Italy; 8 Department of Clinical, Special and Dental Sciences, Università Politecnica delle Marche, 60121 Ancona, Italy; 9 Department of Diagnostic Imaging, Area of Cardiovascular and Interventional Imaging, Abruzzo Health Unit 1, 67100 L’Aquila, Italy; 10 Diagnostic Imaging Department, Villa Scassi Hospital-ASL 3, 16149 Genoa, Italy; 11 Department of Biotechnological and Applied Clinical Sciences, University of L’Aquila, 67100 L’Aquila, Italy; 12 Division of Radiology, Istituto Nazionale Tumori IRCCS Fondazione Pascale—IRCCS di Napoli, 80131 Naples, Italy

**Keywords:** radiomics, diagnosis, staging, recurrence, intrahepatic cholangiocarcinoma

## Abstract

Background: This paper offers an assessment of radiomics tools in the evaluation of intrahepatic cholangiocarcinoma. Methods: The PubMed database was searched for papers published in the English language no earlier than October 2022. Results: We found 236 studies, and 37 satisfied our research criteria. Several studies addressed multidisciplinary topics, especially diagnosis, prognosis, response to therapy, and prediction of staging (TNM) or pathomorphological patterns. In this review, we have covered diagnostic tools developed through machine learning, deep learning, and neural network for the recurrence and prediction of biological characteristics. The majority of the studies were retrospective. Conclusions: It is possible to conclude that many performing models have been developed to make differential diagnosis easier for radiologists to predict recurrence and genomic patterns. However, all the studies were retrospective, lacking further external validation in prospective and multicentric cohorts. Furthermore, the radiomics models and the expression of results should be standardized and automatized to be applicable in clinical practice.

## 1. Introduction

Cholangiocarcinoma (CCA) is the liver’s second most common primary malignancy [1,2]. Due to the increasing incidence of CCA, several studies have focused on improving the diagnosis, prognosis, and treatment of patients [3]. CCA diagnosis is routinely achieved through serum markers (CA 19-9, CEA) and radiologic imaging, but in atypical cases, differential diagnosis can be still challenging, so biopsy remains the only tool for definitive diagnosis [4].

Intrahepatic cholangiocarcinoma (ICC) is the most common type of cholangiocarcinoma, and according to pathological classification, it is categorized as mass forming, periductal infiltrating, or intraductal growing [5]. Among the different subtypes, the mass-forming subtype represents 78% of cases [5,6]. Its main morphological pattern is abundant stromal fibrosis, which also influences its radiological imaging behavior [7,8].

**Figure 1:**
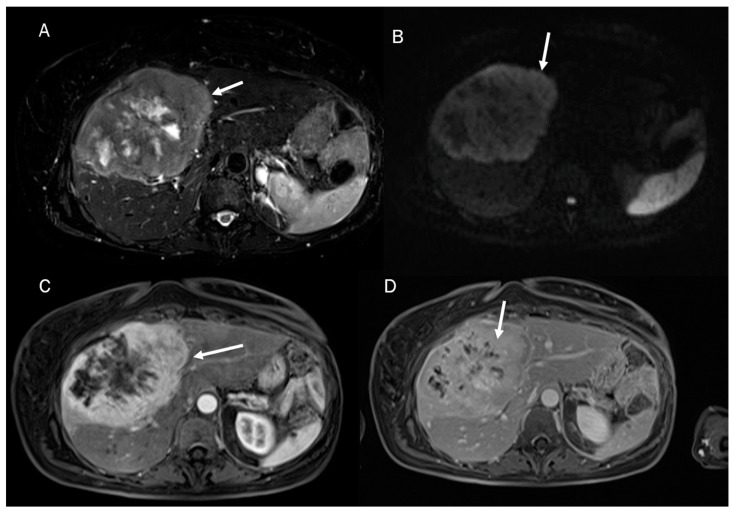
Combined hepatocellular cholangiocarcinoma MRI assessment: the lesion (arrow) shown in T2-W sequence (**A**); targetoid appearance, with restricted diffusion in b800 s/mm^2^ (**B**); and progressive contrast enhancement during contrast study (arterial phase (**C**) and portal phase (**D**)).

Despite the challenging nature of this task, conducting a differential diagnosis between ICC and other liver lesions, especially concerning HCC and combined hepatocellular cholangiocarcinoma (Figure 1), is mandatory to conduct appropriate treatment planning [9,10]. To help radiologists and clinicians, several authors have proposed radiomics models to better define tumor characteristics and disease progression [11,12].

Radiomics belongs to the wider field of artificial intelligence (AI). The most common AI tools are based on machine learning and statistical analysis, while deep learning and neural network represent the most frequent subset [13,14]. The most relevant drawback of machine-learning (ML) AI is the need for a considerable number of data to train the program, so another AI pathway, known as formal methods (FMs), is slowly becoming a reliable tool. This pathway does not need a large sample of images because it is not based on a training set. FMs are defined based on pre-defined rules built on clinical features turned into a numeric and informatic code [15,16,17,18]. The machine-learning approach is the most commonly used. It is capable of learning a large amount of information, so it is gaining even more diffusion in many fields beyond radiology, like nuclear medicine and clinical fields [19,20]. In order to train the most reliable radiomics model, ML was applied to the ultrasound (US) Computed Tomography (CT) or Magnetic Resonance Imaging (MRI) gold-standard protocols already in use for diagnosis, staging, or follow-up in current clinical practice [20,21,22,23]. Radiomics has also demonstrated dependable results differentiating benign from malignant pathologies in many fields whenever treatment strategies could have been radically different [24,25].

Nowadays, radiomics applications are primarily common in oncology concerning neurological, breast, pulmonary, abdominal, and pelvic diseases [26,27,28]. However, the COVID-19 pandemic asked for the earliest diagnosis [29,30]. In this emergency context, radiomics had the chance to demonstrate its efficacy and feasibility; it improved diagnostic accuracy, answering a wide number of clinical questions, and it did so outside of referral centers due to the limitations on mobility for sanitary reasons [31,32,33,34]. At the same time, its limits became evident per a small number of studies focused on its explainability, while in clinical practice, many tools required a long work time and the necessity of standardization of the analysis [35,36,37,38]. In the large field of diagnosis, radiomics has been first developed to classify different lesions in order to avoid further more invasive exams [39,40]. Subsequent applications of radiomics have been found in predicting tumor grade and helping radiologists detect challenging precancerous syndromes [41,42,43,44,45,46]. The latest fashion in AI application is represented by the role of radiomics in the prediction of response to surgical or medical treatment in cancer patients [47,48,49,50,51]. In this way, radiomics can be used to speculate as to the risk category classification of patients and to predict patient overall survival and risk of complication [52,53,54,55,56,57,58,59,60].

Hepatobiliary and pancreatic cancers have been deeply investigated through AI methods [61]. Authors have aimed to recognize primitive and metastatic lesions or to distinguish benign lesions from malignant ones when the limits of conventional imaging techniques did not allow a proper differential diagnosis [62,63]. The most frequent application of radiomics has involved CT scan; however, growing interest has been shown regarding integrated imaging [64,65,66,67].

As depicted above, ICC represents a natural field of interest of radiomics tools due to its ability to exhibit atypical behavior that makes, in some cases, the diagnosis and the subsequent treatment strategy very challenging [4,5,6]. The aim of this review is to report the results of several studies and the real application of radiomics in clinical practice in the large field of diagnosis. The included studies address the following main topics: the prediction of recurrence, the assessment of lymph node status, and the prediction of tumor mutation.

## 2. Methods

We searched the PubMed database (US National Library of Medicine, http://www.ncbi.nlm.nih.gov/PubMed accessed on 15 October 2022) using the subsequent keywords: (((artificial intelligence) OR (radiomics) OR (convolutional neural networks) OR (machine learning) OR (radiomic) OR (deep learning) OR (ultrasomics)) AND ((cholangiocarcinoma) OR (cholangiocellular carcinoma) OR (biliary tumor) OR (Klatskin) OR (hepatocellular cholangiocarcinoma) OR (Combined hepatocellular cholangiocarcinoma)) AND (“English” [Language])).

Papers had to have been published no earlier than October 2022. Articles were first chosen based on title and abstract, but a review of the available full text was necessary to definitely include the article. Clinical studies (retrospective analysis, case series, prospective cohort study) were reviewed. Case reports, reviews, comments, or letters to editors were excluded.

## 3. Results

We recognized 238 pertinent papers. We narrowed down to 89 papers based on a review of titles and abstracts. Then we narrowed further to 61 full-text articles concerning the improvement of diagnosis and treatment strategy.

Articles first excluded for title and abstract were reviews or case reports, or they did not address ICC. Full-text articles excluded did not clearly explain methods and results about diagnosis, recurrence, and staging of ICC.

**Figure 2:**
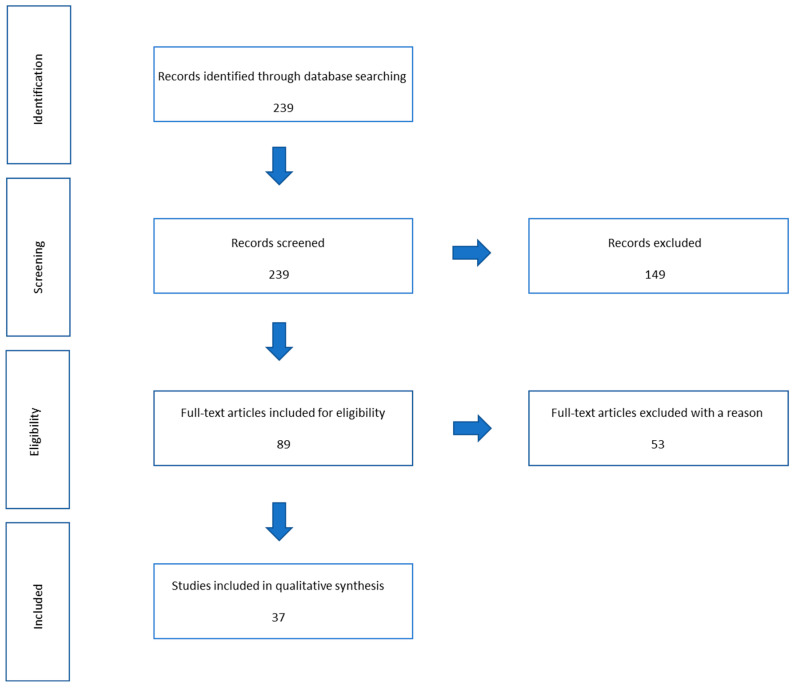
Flowchart of included and excluded studies.

A total of 34 clinical studies, concerning diagnosis, recurrence, and staging, were assessed in this narrative review. The reference flow is summarized in the study flow diagram (Figure 2).

### 3.1. Ultrasound

Ultrasound is an inexpensive, non-invasive imaging tool that is not based on X-ray sources [68,69,70,71,72]. Its plasticity allows operators to best manage uncompliant patients [73,74,75,76]. To overcome several US limits, and to achieve a non-invasive diagnosis, in the last decades, a new technique has been developed mostly based on contrast-enhanced ultrasound (CEUS), or even on shear-wave elastography combined with a CEUS algorithm [77].

CEUS is now used to study large vessel flows and the microcirculation and US behavior of oncological lesions, which might be very helpful for differential diagnosis between benign and malignant tumors [78]. Liver lesions contrast study includes: (1) the arterial phase, which starts at 10–20 s and ends 30–45 s after contrast injection; (2) the portal venous phase, which lasts from 30–45 s to 2 min after contrast agent injection; and (3) the delayed phase, which lasts from 4 to 6 min after the contrast injection [79].

**Figure 3:**
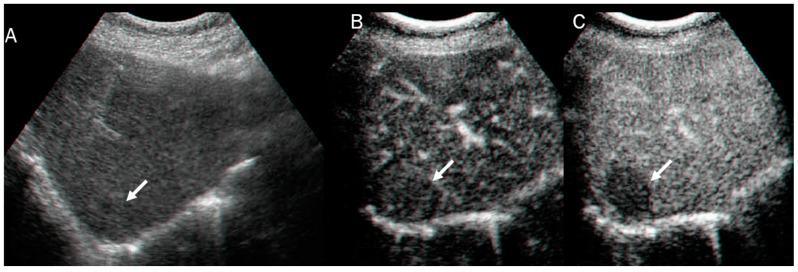
US and CEUS assessment of ICC: On US (**A**), the lesion (arrow) shows iso-hypoechoic pattern compared to hepatic parenchymal. During arterial phase (**B**), the lesion shows arterial hyperenhancement (arrow), with washout (arrow) in portal phase (**C**).

In US studies, mass-forming ICC occurs as a large non-encapsulated mass with lobulated or variable shape. It can also be associated with liver capsule retraction and dilated peripheral bile ducts [80]. With respect to its pathomorphological characteristics (necrosis, fibrosis, and tumor growth), ICC can show a heterogeneous basal-US echogenicity pattern [81]. During CEUS assessment, ICC could show hyperenhancement during the arterial phase (Figure 3) with washout. According to several authors, ICC washout at its earliest stage is comparable to HCC, and this finding should guide a correct diagnosis [82,83,84].

As shown in international guidelines, the differential diagnosis between HCC and ICC can be challenging, especially in non-cirrhotic patients, where the typical radiological pictures of mass-forming ICC might be very similar to pictures of HCC enhancement pattern, requiring liver biopsy to achieve the correct diagnosis [85]. CEUS achieved a reliable sensitivity in differentiating ICC from HCC of 0.92 with a pooled specificity of 0.87 [86,87]. Considering that the diagnostic performance of CEUS is very changeable as the diagnostic technique is operator-dependent, ultrasomics surely could make the US exams more repeatable and reliable, standardizing the technique; in fact, in the comparison studies between radiologists and ultrasomics model performance, the latter achieved a better sensitivity and global accuracy [87]. Ultrasomics has been proven to be useful in the early diagnosis, preoperative grading prediction, therapeutic efficacy evaluation, and prognosis evaluation of several tumors [88,89,90,91,92,93].

Regarding the differential diagnosis of liver lesions, ultrasomics-based studies achieved a good accuracy in the validation or test set [89,90]. In a study by Peng et al., patients were classified into 3 groups: 89 ICC, 531 HCC, and 48 combined hepatocellular cholangiocarcinoma (cHCC-ICC). The overall performance of the radiomics model in identifying different histopathological subtypes was moderate, with AUC values of 0.854 (training cohort) and 0.775 (test cohort) in the HCC vs. non-HCC model and 0.920 (training cohort) and 0.728 (test cohort) in the ICC vs. cHCC-ICC radiomics model [89]. Ren et al. assessed two subgroups: HCC and non-HCC. The combined (clinical + radiomics) model achieved the highest accuracy in the external validation set, with an AUC of 0.874, a sensitivity of 0.900, a specificity of 0.857, and an accuracy of 0.868 [90]. Li et al. [91] compared the diagnostic performance between the ultrasomics-based model and the CEUS Liver Imaging Reporting and Data System (LI-RADS) v2017. The ultrasomics model achieved a better sensitivity than LI-RADS (90.6% vs. 81.3%) and a better accuracy (90% vs. 83%). No differences were found on specificity and AUC. Although the results were encouraging, the ability to differentiate ICC from HCC remains low [92].

The ultrasound radiomic signature was also helpful to predict the biological characteristics of ICC. Peng et al. showed moderate efficiency in predicting the biological behaviors of 128 ICC, evaluating six pathological features. They reached the best results predicting ki67, VEGF, and CK7 (0.848, 0.864, and 0.789, respectively). Ki67 also achieved the best sensitivity, at 0.957, but a specificity of 0.500 [93].

The results obtained by the ultrasomics model are still related to clinical data and may be influenced by the operator who acquires the images [88]. Concerning differential diagnosis, the ultrasomics model can improve the diagnostic accuracy of radiologists in the characterization of liver lesions, especially in cases of underlying liver disease [94].

Although the results of the ultrasomics model compared with LI-RADS were encouraging, the difference between the two scores in the ability to differentiate ICC from HCC was not significant [92]. The data obtained on liver ultrasomics were similar to those obtained on thyroid, breast, or kidney ultrasomics [95,96,97,98,99,100,101].

Radiogenomics is an emerging research field that aims to correlate imaging features with the underlying genes or mutated genes [102,103]. Though most of these studies have been based on CT or MRI radiomics tools, ultrasomics has also been used especially for breast cancer, not only for the diagnosis of the lesions, but also for the prediction of the molecular subtype, with a reported accuracy of 95% [104,105,106,107].

As for the other applications of ultrasomics, for liver cancer, it is also possible to identify the limitations that currently hinder its translation into clinical practice, as there is a need for prospective multicentric studies and for automatizing the expression of results.

### 3.2. Computed Tomography

**Figure 4:**
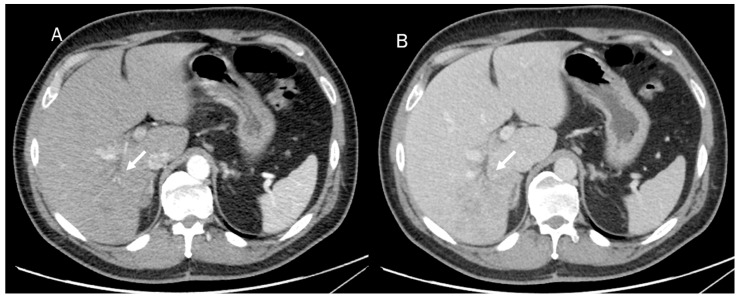
CT assessment of ICC (arrow) during arterial (**A**) and portal (**B**) phase of contrast study. The lesion (arrow) shows an infiltrative pattern with biliary tree dilatation.

Mass-forming ICC usually appears at basal CT as a hypodense lesion presenting either a well-defined border or an infiltrative pattern without its own capsule (Figure 4). It is associated with heokpatic capsule retraction in about 20% of cases [108,109]. After contrast administration, the nodule shows initial peripheral rim enhancement, followed by progressive and concentric filling with contrast material as an effect of fibrosis, which is slow to enhance but retains the intravenous contrast agent [110,111].

Even though the specificity of conventional CT in characterizing lesions may appear comparable to CEUS, CT is still mandatory in pre-surgical settings to value lesion relationships with major vessels and to quantify its volume [112].

In the era of technologies innovation, dual-energy CT (DECT) based on iodine quantification can serve as a tool to improve the diagnostic accuracy of the standard CT for the differentiation of ICC and HCC [113].

Regardless of the technology used, CT evaluation of cirrhotic livers remains a challenge for radiologists due to the development of fibrous and regenerative tissue that causes the distortion of normal liver parenchyma [114,115]. This can cause a misdiagnosis of ICC, HCC, and cHCC-ICC or even hinder differentiation between malignant and benign lesions [116,117]. In the literature, these misleading patterns were reported in 5–10% of patients [118,119].

For all these reasons, several studies have proposed AI models based on CT features to correctly classify liver lesions, avoid more invasive procedures, and choose the correct timing of further radiation doses [119,120,121]. The majority of the enrolled studies focused on diagnosis of ICC, cHCC-ICC, and HCC, and they did not include any rare liver disease [122,123,124]. Reviewing these studies, it was possible to conclude that all the radiomics tools are based on machine learning. The sample of patients included and analyzed strongly conditioned results; therefore, studies with a small sample of patients/groups needed further external validation [125,126,127,128,129,130,131,132,133]. However, despite the technical limitations associated with the need for a manual definition of ROIs in more than one contrast phase, radiomics models allowed promising results to be obtained. The two studies that were considered more reliable due to the larger patient sample are those of Zhou et al. [131] and Yasaka et al. [132]. Zhou et al. assessed 616 nodules, including malignant lesions (HCC, ICC, and metastasis) and benign lesions (hemangioma, focal nodular hyperplasia, and cyst) using a deep-learning approach (accuracy of 73.4%) [131]. Similar results were obtained by Yasaka et al. on 460 patients classified as having liver lesions using deep-learning (CNN) applied to CT images in the arterial and delayed phases [132]. With regard to rare hepatic lesions, an interesting study analyzed an ML approach in differential diagnosis between hepatic lymphoma (HL) and ICC [133]. The model showed a good performance and high accuracy; however; these results are less reproducible since the HL group was composed of 28 patients. Further study with external validation is expected [133].

With regard to the ICC risk assessment, recently, intrahepatic lithiasis (IHL) has been related to the development of ICC, with a conversion rate estimated between 2.4 and 13.0% [134,135,136]. It is very difficult for clinicians and radiologists to identify ICC hidden behind IHL because there are no specific symptoms or radiological features [137,138,139,140,141,142,143]. Tissue biopsy is not routinely recommended, and its negative result does not exclude the presence of malignancies [137]. Therefore, the current diagnostic accuracy of IHL-ICC is low, generally ranging from 30 to 65%. Xue et al. assessed 131 at-risk patients, showing a good performance by using a rad-score combined with a clinical-radiological model [136].

With regard to the ability of radiomics in the prediction of recurrence after treatment, several studies used preoperative or post-operative features [144,145,146,147,148,149,150]. Jolissant et al. predicted ICC recurrence 1 year after surgical treatment by building a model on texture features (TFs) extracted from the liver, from the tumor, and from the future liver remnant (FLR) on preoperative images [145]. Patients with early recurrence had a larger tumor size and a higher rate of lymph node metastasis (LNM) but were not more likely to have multifocal disease (21.4% vs. 17.4%, *p* = 0.643). The combined model with texture features and tumor size achieved the highest AUC of 0.84 (95% CI 0.73–0.95) in predicting recurrence in the validation cohort [145]. Similar results were obtained by Zhu et al. [147]. Their model was built on a logistic regression that combined preoperative and pathological features (solitary, size, differentiation, membrane invasion, portal venous phase CT value mean, equilibrium phase CT value mean, energy ap, inertia ap, percentile50th-portal phase value) and showed high diagnostic performance in terms of sensitivity (0.818) and specificity (0.909) [147].

The study by Chu et al. was the first study based on surgical technique with the aim of avoiding futile resection in high-risk-recurrence ICC. They achieved a sensitivity of 0.846 and a specificity of 0.771 in the validation cohort, comparable with previous studies. Futile resections are related to the impossibility of performing an R0 resection due to a discrepancy between preoperative evaluations and intraoperative findings. Because 16% of patients risk futile resection, the study had a clear application in clinical practice [150].

Proper patient management requires a correct disease stage assessment and a critical lymph nodes assessment to plan the correct treatment strategy and surgical approach. At present, the limit of conventional imaging for a pre-surgical-nodes-involvement evaluation is known, so great attention is being shown toward radiomics [140,151,152]. Ji et al. [140] and Zhang et al. [152] proposed a methodology to predict lymph node (LN) metastasis of ICC and to determine its prognostic value, obtaining similar results on a validation cohort [140,152].

Biological characteristics related to poor prognosis were also evaluated [153,154]. Isocitrate dehydrogenase (IDH) is frequently mutated in ICC (10–28%) and holds great prognostic significance. Zhu et al. predicted this mutation through CT-radiomics features (a global accuracy of 0.863 and an AUC of 0.813) [155].

Although differential diagnosis with high accuracy is considered a hot topic for radiomics studies, prospective and multicentric studies are needed to validate the models. In fact, the sample of each group impacts the reliability achieved by the ML tool [125,126,127,128,129,130]. The models proposed are built on analyses with huge variability and need to be standardized. Furthermore, CT image texture analysis needs the definition of a precise ROI, excluding vessels, bile ducts, and colliquative areas or calcification; therefore, to validate the model in clinical practice, an automatization of the ROI or VOI definition is mandatory to reduce the work time [135,136,137,138,139,140].

In addition, the prediction of the lymph nodes involved can have effects in clinical practice. In fact, early recurrence and involvement of lymph nodes impact the choice of liver transplantation for unresectable ICC despite medical therapy [156,157,158]. Precision medicine, and consequently precision oncology, like precision surgery, should be based on these features not immediately visible to the human eye [159,160,161,162]. Therefore, there is a need for advanced technologies such as radiomics, target therapy, and minimally invasive liver surgery [163,164,165,166,167,168,169,170,171,172,173,174,175,176].

### 3.3. Magnetic Resonance Imaging

In the current clinical practice, MRI is performed in association with CT as standard of care to complete the study pre-treatment of cholangiocarcinoma, to evaluate the invasion of bordering structure or soft tissue, bile duct, and blood flow and the vascular morphology in the portal venous system [177,178,179,180].

**Figure 5:**
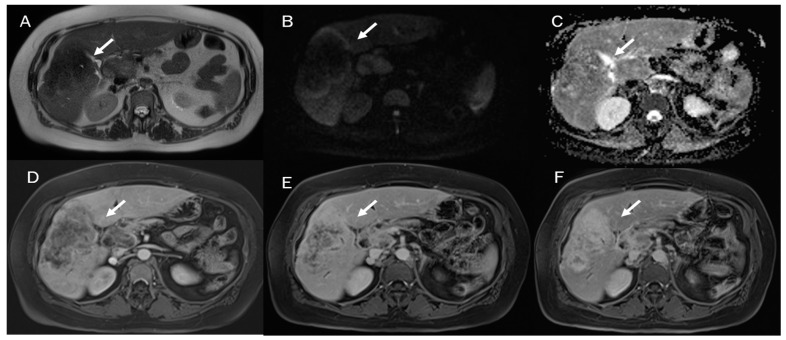
ICC MRI assessment. The lesion (arrow) shows hypointense signal in T2-W sequence (**A**) due to fibrotic tissue, with targetoid appearance in DWI (**B**) and ADC map (**C**) and progressive contrast enhancement during arterial (**D**), portal (**E**), and delay (**F**) phases of contrast study.

**Figure 6:**
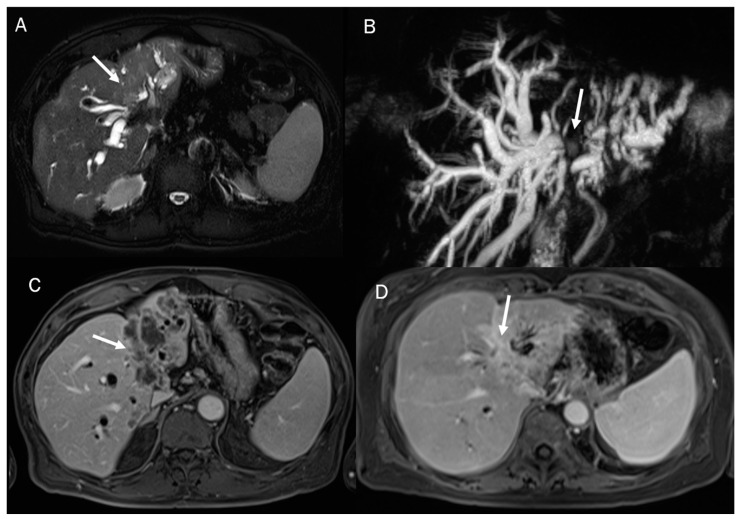
MRI assessment of periductal-infiltrating CCA. The lesion (arrow) shows hyperintense signal in T2-W (**A**), causing biliary tree dilatation in cholangiography sequences (**B**). During arterial phase (**C**), the lesion causes hyperenhancement of surrounding liver parenchymal, showing a progressive contrast enhancement in portal phase (**D**).

In MRI imaging, ICC presents typical features as capsular retraction adjacent to the tumor. In T1-W sequences, the lesion appears with a targetoid aspect or hypointense signal. While most of the lesions also appear targetoid (Figure 5) in T2 sequences, some can show hyperintense signals. After contrast administration in the arterial phase in ICC, it is possible to identify the peripheral rim hyperenhanced. In the portal phase, the lesion slowly increases its entire enhancement (Figure 5 and Figure 6) [181,182,183,184].

A hepatocyte-specific contrast agent, gadolinium ethoxybenzyl diethylenetriamine pentaacetic acid (Gd-EOB-DTPA), enhances the blood pool and is hepatocyte specific, since it is taken up by hepatocytes and excreted into the biliary tract (EOB phase). Approximately 50% of the administered dose of Gd-EOB-DTPA is taken up by normal hepatocytes and subsequently excreted into the biliary tract, while the remaining 50% is excreted via the kidney. Hepatocellular uptake is considered to represent passive diffusion mediated by organic anion transporter polypeptide 1 (OATP1), which is expressed on the hepatocyte membrane. Gd-EOB-DTPA-enhanced MRI may offer a breakthrough for the diagnosis of liver tumors. In the EOB phase, ICC has a hypointense signal, although considering fibrotic structure, part of the administrated dose could be detected inside the lesion. In addition, the possibility of using this type of agent to assess the ICC microenvironment could help in the treatment decision phase [6,181]. The CCA tumor microenvironment is a dynamic environment consisting of authoritative tumor stromal cells and an extracellular matrix where tumor stromal cells and cancer cells can thrive. CCA stromal cells include immune and non-immune cells, such as inflammatory cells, endothelial cells, fibroblasts, and macrophages. Likewise, the CCA tumor microenvironment contains abundant proliferative factors and can significantly impact the behavior of cancer cells. Through abominably intricate interactions with CCA cells, the CCA tumor microenvironment plays an important role in promoting tumor proliferation, accelerating neovascularization, facilitating tumor invasion, and preventing tumor cells from organismal immune reactions and apoptosis [179,180,181].

As for CT, the MRI-based tool achieved high accuracy in differential diagnosis among liver lesions, especially HCC and ICC. Most of the studies were conducted through machine-learning-based tools [185].

Concerning differential diagnosis, the sensitivity of gadoxetic acid-enhanced MRI should not be influenced by underlying chronic liver disease, but rather by hypervascular tumors [185]. Radiomics applied to multiphasic MRI achieved great results. A large training cohort composed of 494 lesions and a test cohort of 60 lesions achieved a sensitivity of 88% in classifying the lesions [186,187]. These results were supported by Zhou et al. (AUC of 0.80), who included ICC and cHCC-ICC [188].

In addition, in T2-W MRI images, Huang et al. proposed a methodology able to differentiate HCC from ICC in 174 patients (113 cases of HCC and 61 cases of mass-type ICCA). The AUC of the radiomics nomogram was 0.97 in the training group and 0.95 in the validation group. The results are comparable to those obtained with post contrast sequences [189].

Recent advances in machine learning brought an automatization of the model to accelerate workflow, enhance performance, and increase the accessibility of AI to clinical researchers [190]. Hu et al.’s study through auto ML achieved an accuracy similar to that of manual optimization with a sensitivity and specificity comparable to that of radiologists. However, automated ML needs to be improved on the diagnosis of LR-M of LI-RADS and needs additional features to be implemented [190,191].

The studies conducted on MRI images achieved a stronger diagnostic power than those on CT images, but prospective and multicentric studies are needed [131,132,187,188].

The theme of recurrence was also explored through MRI. The results achieved were comparable to the ones achieved by CT images analysis [192,193,194,195,196]. In the study by Xu et al., to predict early and late recurrence, features were extracted from the intratumoral and peritumoral area. The combined model obtained an AUC of 0.852. The early recurrence was also predicted by using post-contrast sequences, combining radiological features and immunohistochemical markers (AUC of 0.949, sensitivity of 0.875, and specificity of 0.774) [192].

With regard to nodal involvement, Xu et al. tested a model to identify lymph node metastasis in 106 patients with ICC, showing a good discrimination in separating patients with nodal metastases and without nodal involvement LNM and non-LNM (AUC: the training group: 0.842 vs. 0.788; the validation group: 0.870 vs. 0.787) [194].

With regard to prognostic assessment, several authors assessed the ability of radiomics in determining molecular characteristics, mutational status, and microvascular invasion (MVI).

Zhang et al. proposed a model to investigate the expression of PD-1/PD-L1 in ICC. The model was built on MRI images in the arterial and portal vein phases of 98 patients. The highest area under the curves of the models predicting PD-1 and PD-L1 expression was 0.897 and 0.890, respectively [197]. Zhou et al. developed a model on DCE-MRI to predict MVI in mass-forming ICC patients. Larger tumor size and higher radiomics scores were positively correlated with MVI in both the training cohort (*p* < 0.001, <0.001, respectively) and the validation cohort (*p* = 0.008, 0.001, respectively). The radiomics signature showed optimal prediction performance in validation cohorts (AUC of 0.850) [198]. Similar results were obtained by Qian et al. (AUC of 0.819 in the test cohort) for the MVI prediction model, which incorporated tumor size and intrahepatic duct dilatation [199].

Regarding MRI and radiomics, although the results are promising, several questions remain open regarding the sample under examination in terms of population homogeneity and external validations. The use of study protocols and different equipment make the results not very reproducible. In addition, some authors proposed an analysis based on DCE-MRI, where it concerns studies of CE-MRI, since they assessed specific contrast phases as arterial or portal. The optimization of the protocols could certainly lead to a greater robustness of the results.

## 4. Conclusions

Advances in artificial intelligence must be interpreted with caution. Virtually all studies about AI were made retrospectively, and more research is needed to make sure than the use of AI provides equivalent results in real-world prospective studies [200].

Many performing models have been developed to make differential diagnosis easier for radiologists and offer the chance to predict recurrence and genomic patterns. However, we have to underline that all the studies were retrospective, lacking further external validation in prospective and multicentric cohorts. Furthermore, the radiomics models and their expression of results should be standardized and automatized to be applicable in clinical practice.

## Data Availability

All data are reported in the manuscript.
